# Supplementary material for: Seminoma and Embryonal Carcinoma Footprints Identified by Analysis of Integrated Genome-Wide Epigenetic and Expression Profiles of Germ Cell Cancer Cell Lines
Source: PLoS One. 2014 Jun 2;9(6):e98330. doi: 10.1371/journal.pone.0098330 (PMC4041891; doi:10.1371/journal.pone.0098330)
Supplement: File S1 — ZIP file containing DMRforPairs output for significant regions. Please start from the html files. (ZIP) [file pone.0098330.s008.zip › figures/9789.pdf]

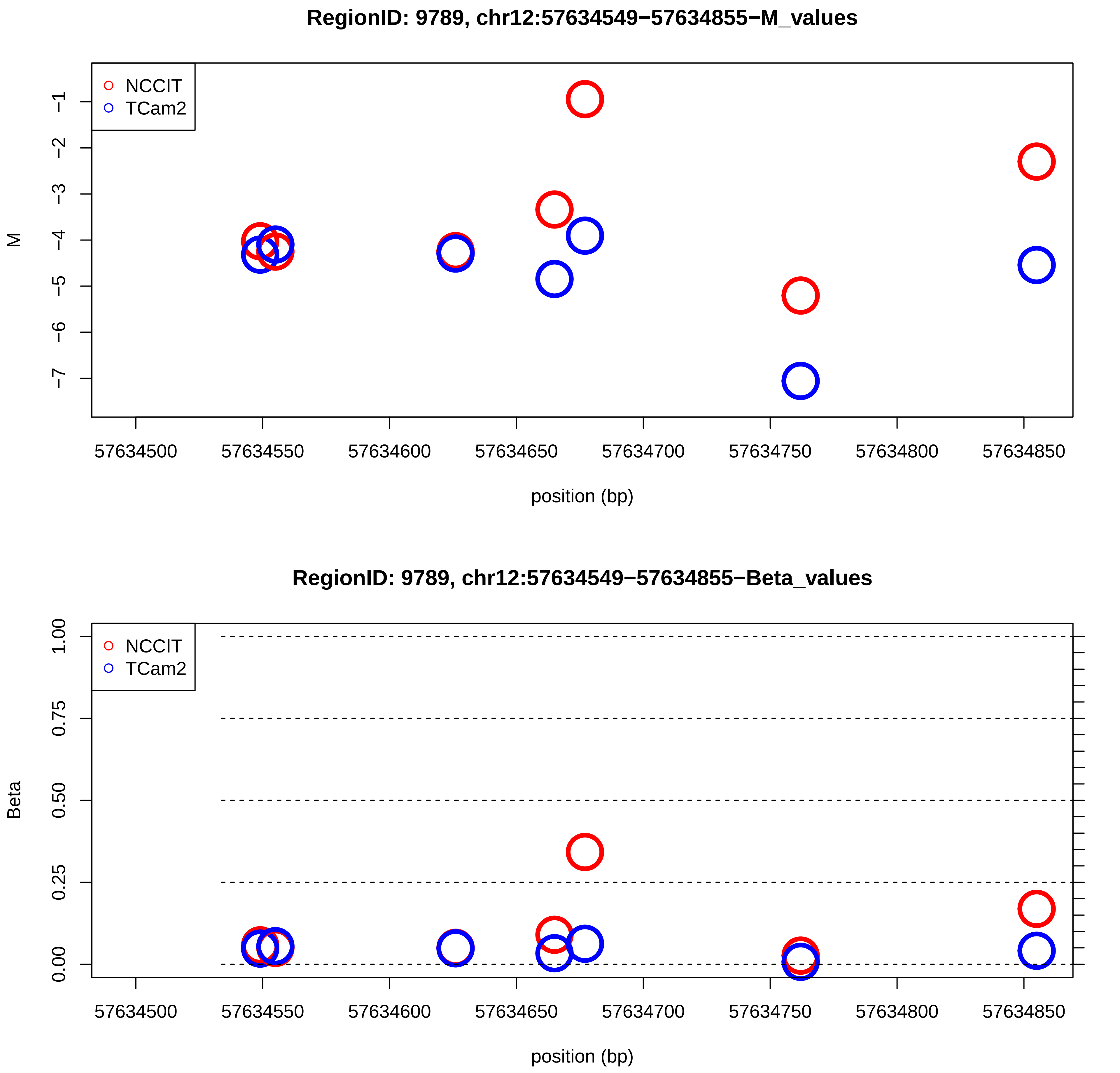
RegionID: 9789, chr12:57634549−57634855−M_values
NCCIT
TCam2
M
−1
−2
−3
−4
−5
−6
−7
57634500
57634550
57634600
57634650
57634700
57634750
57634800
57634850
position (bp)
RegionID: 9789, chr12:57634549−57634855−Beta_values
NCCIT
TCam2
Beta
0.00
0.25
0.50
0.75
1.00
57634500
57634550
57634600
57634650
57634700
57634750
57634800
57634850
position (bp)
